# Disruption of β-catenin-mediated negative feedback reinforces cAMP-induced neuronal differentiation in glioma stem cells

**DOI:** 10.1038/s41419-022-04957-9

**Published:** 2022-05-24

**Authors:** Zhijie Chen, Yingqian Zhong, Jiehong Chen, Shuxin Sun, Wenfeng Liu, Yu Han, Xincheng Liu, Cui Guo, Depei Li, Wanming Hu, Peiyu Zhang, Zhuopeng Chen, Zhongping Chen, Yonggao Mou, Guangmei Yan, Wenbo Zhu, Wei Yin, Ke Sai

**Affiliations:** 1 Department of Neurosurgery/Neuro-oncology, Sun Yat-sen University Cancer Center, Guangzhou, 510060 China; 2 State Key Laboratory of Oncology in South China, Collaborative Innovation Center for Cancer Medicine, Sun Yat-sen University Cancer Center, Guangzhou, 510060 China; 3 Department of Neurosurgery, The Third Affiliated Hospital of Sun Yat-sen University Lingnan Hospital, Guangzhou, 510530 China; 4 Department of Pharmacology, Zhongshan School of Medicine, Sun Yat-sen University, Guangzhou, 510080 China; 5 Department of Pancreas Center, Guangdong Provincial People’s Hospital, Guangdong Academy of Medical Sciences, Guangzhou, 510080 China; 6 Department of Pathology, Sun Yat-sen University Cancer Center, Guangzhou, 510060 China

**Keywords:** Cancer stem cells, Drug development, CNS cancer

## Abstract

Accumulating evidence supports the existence of glioma stem cells (GSCs) and their critical role in the resistance to conventional treatments for glioblastoma multiforme (GBM). Differentiation therapy represents a promising alternative strategy against GBM by forcing GSCs to exit the cell cycle and reach terminal differentiation. In this study, we demonstrated that cAMP triggered neuronal differentiation and compromised the self-renewal capacity in GSCs. In addition, cAMP induced negative feedback to antagonize the differentiation process by activating β-catenin pathway. Suppression of β-catenin signaling synergized with cAMP activators to eliminate GSCs in vitro and extended the survival of animals in vivo. The cAMP/PKA pathway stabilized β-catenin through direct phosphorylation of the molecule and inhibition of GSK-3β. The activated β-catenin translocated into the nucleus and promoted the transcription of APELA and CARD16, which were found to be responsible for the repression of cAMP-induced differentiation in GSCs. Overall, our findings identified a negative feedback mechanism for cAMP-induced differentiation in GSCs and provided potential targets for the reinforcement of differentiation therapy for GBM.

## Introduction

As one of the most prevalent primary intrinsic brain tumors in adults, glioblastoma multiforme (GBM) is a universally lethal cancer with a median survival of less than 2 years [1]. GBMs contain strikingly heterogeneous cellular populations with diverse biological features and transcription profiles [2]. It has been well recognized that GBMs are organized as a hierarchy containing a sub-population of cells that phenotypically and functionally resemble neural precursor cells [3]. This small pool of stem-like glioma cells, namely, glioma stem cells (GSCs), are capable of self-renewal and propagation by symmetric and asymmetric divisions [4, 5]. GSCs play a critical role in mediating therapeutic resistance and are considered as a promising target [6, 7].

Differentiation therapy with cytokines or chemical agents is a promising alternative strategy to treat cancers by forcing self-renewed tumor cells to a mature post mitotic state [8]. The notable success of all-trans retinoic acid (ATRA) is a paradigm for differentiation therapy. Pioneering observations demonstrate ATRA induces terminal differentiation of acute promyelocytic leukemia ex vivo and leads to durable clinical remission when combined with arsenic trioxide [9]. Differentiation therapy has also been extensively investigated in solid tumors. Antibodies targeting PTPRK-RSPO3 fusion promote differentiation and abolish stem-cell compartment in colon cancers [10]. Inhibition of lysine-specific histone demethylase 1A (LSD1A) reprograms the transcriptional profiles and results in cell cycle arrest as well as differentiation in Merkel cell carcinoma [11]. In gliomas, delivery of bone morphogenetic protein 4 (BMP4) reduces the GSC pool and elicits the expression of markers of neuronal lineage [12]. The secondary messenger, cyclic AMP (cAMP), has the capacity to drive glioma cells to differentiation. Activation of the cAMP/PKA/CREB pathway by cholera toxin inhibits proliferation and induces differentiation towards mature astrocytes in C6 glioma cells [13]. Additionally, cAMP reverses the Warburg effect by enhancing mitochondrial biogenesis and directs differentiation of human glioma cells through CREB-PGC1α signaling [14]. However, glioma cells fail to undergo full commitment in response to differentiation cues, which is mainly due to the complicated oncogenic signaling in gliomas [15, 16]. As a result, identification of key molecules responsible for the negative regulation of differentiation is crucial to enhance the efficacy of differentiation therapy. In the current study, we demonstrate that cAMP induces neuronal differentiation in GSCs and simultaneously stabilizes and activates β-catenin. The activation of β-catenin conversely antagonizes the differentiation process in GSCs. Knock-down or pharmacological inhibition of β-catenin synergizes with cAMP activators to eradicate the GSC population in vitro and retards the tumor growth in vivo. We identify that APELA and CARD16 are downstream effectors of β-catenin that are responsible for the suppression of cAMP-induced differentiation in GSCs. Our findings unveil a negative feedback mechanism in cAMP-induced differentiation and provide promising targets to reinforce differentiation therapy for gliomas.

## Materials and methods

### Cell culture and reagents

GSC1 and GSC11 cell lines were derived from human GBM tumor samples and were characterized as reported previously (also in Supplementary Fig. S1) [17–20]. Briefly, freshly resected GBM tissues were cut into small pieces, washed three times in DMEM/F12 medium (Gibco, Carlsbad, CA, USA), digested with trypsin (Gibco), triturated mechanically with surgical scissors, and filtered through a cell strainer (BD Biosciences, Franklin Lakes, NJ, USA). The erythrocytes were removed with a red cell lysis buffer (Tiangen, Beijing, China). Tumor cells were then washed repeatedly with DMEM/F12 medium and were cultured in serum-free DMEM/F12 medium supplemented with 2% B27 (Gibco), 20 ng/mL basic fibroblast growth factor (bFGF, Gibco), and 10 ng/mL epidermal growth factor (EGF, Gibco). These two cell lines were maintained in a humidified atmosphere at 37 °C under 5% CO2. GSC-spheres were dissociated by using StemPro Accutase Cell Dissociation Reagent (Gibco) for serial passaging or further experiments. For differentiation, we used poly-L-Lysine (PLL) coated 6-well plates (0.5–1 × 10^5^ cells/well) [21]. The following reagents were used in this study: dbcAMP (100 mM, dissolved in double distilled water, #D0627-1G, Sigma-Aldrich, St. Louis, MO, USA), forskolin (20 mM, dissolved in DMSO, #S2449, Selleck Chemicals, Houston, TX, USA), Luteolin (25 mM, dissolved in DMSO, #S2320, Selleck Chemicals), and FH535 (20 mM, dissolved in DMSO, #S7484, Selleck Chemicals).

### Neurosphere formation assay

Dissociated GSCs were seeded in 48-well flat-bottomed plates at a density of 4 × 10^3^ cells per well. GSCs were treated with designated agents. After 72 h incubation at 37 °C in a humidified 5% CO2 atmosphere, three pictures were taken per condition at 40× magnification and at least 10 newly formed neurospheres diameter were measured per condition under microscopy (Nikon Eclipse Ti-U, Plan Fluor 4×/0.13 objective, Tokyo, Japan). Single cells were not included into quantification.

### Limiting dilution assay

Limiting dilution assay was performed as described previously [4, 22]. GSC cells were dissociated and seeded in 96-well plates in 200 μL of serum-free medium with final cell dilutions ranging from 400 cells per well to 1 cell per well in replicates of 10. After 1 weeks, spheres larger than 100 μm in diameter were counted by using inverted microscopy. ELDA software was employed to calculate the self-renewal capacity of GSC cells [23]. The ELDA results were plotted by using *statmod* package in R software.

### Western blot

Soluble proteins from treated cells were harvested and lysed in solution containing 50 mM HEPES (pH 7), 150 mM NaCl, 1.5 mM MgCl_2_, 1 mM EGTA, 100 mM NaF, 10 mM sodium pyrophosphate, 10% glycerol, 1% Triton X-100, 1 mM Na_3_VO_4_, 10 μM pepstatin, 10 μg/mL aprotinin, 5 mM iodoacetic acid, and 2 μg/mL leupeptin. Equal amounts of protein were resolved by SDS-PAGE and transferred to polyvinylidene difluoride membranes (Roche, Basel, Switzerland). The membranes were then probed with the following primary antibodies: CD133 (#64326S, Cell Signaling Technology, Danvers, MA, USA), SOX2 (#23064 S, Cell Signaling Technology), β-catenin (#8480S, Cell Signaling Technology), Ser675-phosphorylated β-catenin (#4176S, Cell Signaling Technology), Ser9-phosphorylated GSK3β (#9336S, Cell Signaling Technology), GSK3β (#5676S, Cell Signaling Technology), PKA (#5842S, Cell Signaling Technology), GAPDH (#Ab103, Vazyme, Nanjing, China), and β-actin (#Ab101-03, Vazyme). Immunoreactivity was visualized by probing with the HRP-conjugated secondary antibody (#7074, Cell Signaling Technology) and detected using the ECL kit (#sc-2048, Santa Cruz, Santa Cruz, CA, USA). Western blot band intensities were quantified through densitometric analysis using ImageJ and normalized the band intensities with the loading control band. Original uncropped WB are given in Supplementary Material.

### Flow cytometry analysis

Flow cytometry (FCM) analysis was performed by using a Gallios flow cytometer (Beckman Coulter). For cell proliferation assessment, the cells were incubated with 10 mM EdU (Sigma-Aldrich) for 2 h, and EdU was chemically conjugated to 50 mM Auto 488 (Sigma-Aldrich) for 1 h and incubated with 1 mg/mL DAPI for another 30 min. For neuronal markers analyses, cells were collected after treatment with vehicle or cAMP activators, and then incubated with 1:100 dilution of MAP2 (#FCMAB318PE, Merk Millipore, Temecula, CA, USA) and 1:100 dilution of TUJ1 (β-Tubulin class III Alexa Fluor 488, #560381, BD Pharmingen, San Diego, CA, USA) for 1 h.

### Immunofluorescence assay

Cells cultured as monolayers were washed with phosphate-buffered saline (PBS), fixed in 4% paraformaldehyde/PBS for 20 min at room temperature, and then permeabilized in 0.2% Triton/PBS for 10 min. After blocking in 2% bovine serum albumin/PBS at room temperature for 30 min, cells were incubated with the following primary antibodies: β-catenin (#8480S, Cell Signaling Technology, 1:250 dilution), MAP2 (#4542S, Cell Signaling Technology, 1:250 dilution), and TUJ1 (β3-Tubulin, #4466 S, Cell Signaling Technology, 1:250 dilution) for 1 h at room temperature or overnight at 4 °C. Then, cells were washed three times with PBS for 10 min each and incubated with secondary antibody (donkey-anti-mouse Alexa 568 or goat-anti-rabbit Alexa 488, Invitrogen; 1:500 dilution in PBS containing 2% bovine serum albumin) for 1 h at room temperature. After washing with PBS, cells were incubated with DAPI for 5 min and then washed three times with PBS and rinsed with ddH2O once. Immunofluorescent imaging was performed on a Nikon A1 confocal microscope. ImageJ software was used to assess immunofluorescence, with thresholds set according to signal intensity. The fluorescence intensity of TUJ1 and MAP2 in approximately 30 GSC cells from random fields was quantified and the mean fluorescence intensity was calculated as $\frac{{{\rm{average}}\;{\rm{fluorescence}}\;{\rm{intensity}} \times {\rm{area}}}}{{{\rm{cell}}\;{\rm{number}}}}$. The fluorescence level of total β-catenin was determined by randomly selecting fields in each image and calculating their average fluorescence. For measuring the nuclear β-catenin mean fluorescence intensity, at least 30 cells were randomly selected, and the average nuclear fluorescent intensity was calculated in nuclear areas outlined by DAPI staining. Background fluorescence (defined as the average fluorescence of the area outside nucleus) was subtracted from the average fluorescence of nucleus for each image.

### Immunohistochemical staining

Brain tissue from mice were quickly dissected and fixed in 4% paraformaldehyde overnight, then dehydrated with ascending grades of ethyl alcohol, and cleaned in xylene and embedded in paraffin, and sectioned at a thickness of 4 μm. Immunohistochemical (IHC) staining was performed as follows. According to the manufacturer’s instructions of the IHC staining kit (#ab80436, Abcam, Cambridge, MA, USA). Slices were placed with antigen retrieval for 30 min, and in 3% hydrogen peroxide for 15 min after the gradients of deparaffinization and hydration. Cooling to room temperature, slices were incubated with indicated primary antibodies against Ki-67 (#9449S, Cell Signaling Technology) and MAP2 (#ab32454, Abcam) overnight at 4 °C, and then incubated with an HRP-conjugated secondary antibody (#7074, Cell Signaling Technology) at room temperature. Washing with PBS three times, slices were stained with a Diaminobenzidine (DAB) substrate–chromogen mixture in turn. Images were captured using an inverted microscope (Eclipse Ti-U, Nikon, Japan). For quantification, the integrated optical density (IOD) of the IHC results were analyzed using ImageJ software and the relative IOD values between groups were compared.

### RNAi experiments

Specific siRNAs targeting CTNNB1, PKA, CARD16, APELA, and MSLN were purchased from RiboBio (Guangzhou, China). siRNAs were transfected using Lipofectamine RNAiMAX (Life Technologies, Carlsbad, CA, USA) with opti-MEM (Life Technologies) following the manufacturer’s instructions.

### qRT-PCR

Total RNA was extracted using TRIzol (Thermo Fisher, Rockford, IL, USA) reagent and the concentration of RNA was detected by a NanoDrop2000 spectrophotometer (Thermo Fisher). RNA was reverse transcribed using a PrimeScrpt RT reagent kit. Specific gene expression was quantified with SuperReal PreMix SYBR Green (Tiangen) using an Applied Biosystems 7500 fast real-time PCR system (Life Technologies). All reactions were performed in triplicate, and human actin was served as the endogenous control. The results were analyzed via the 2^−ΔΔCt^ method.

### Transcriptome data processing and analysis

Raw reads were trimmed to 90 nucleotides in length and were mapped to the UCSC Genome Browser Database hg19 reference genome [24] (http://genome.ucsc.edu/) using TOPHAT (v2.0.11) [25] (http://tophat.cbcb.umd.edu/). Default Tophat settings were used, except for the redefined parameter ‘–mate-inner-dist200’. The inner distance between pair ends was estimated by the PICARD program (http://picard.sourceforge.net). The HTSeq program was used to count mapped reads with the parameters ‘-s no –a 20’ [26]. Genes with less than two reads per million were removed, and other genes were included for further analysis. Gene count normalization and differential expression analysis were performed using the DESEQ package [27].

### Gene annotation and functional enrichment analysis

The Piano package [28] was used for GSEA [29]. Gene sets were retrieved from Gene Ontology (http://geneontology.org/), Kyoto Encyclopedia of Genes and Genomes (KEGG), and the Ingenuity Pathways database (IPA, Ingenuity Systems, http://www.ingenuity.com) [30, 31]. Raw *p* values from the GSEA were corrected for multiple testing by false discovery rate (FDR). Pathways with corrected *p* values of less than 0.05 were considered significant.

Metascape was applied to analyze the functional enrichment [32].

### Subcutaneous and intracranial xenograft models

Mice were housed in a pathogen-free animal facility. The hind flanks of 4-week-old female BALB/c-nu/nu mice were subcutaneously inoculated with 1 × 10^5^ GSCs. After 5 days, palpable tumors developed (50 mm^3^), and the mice were randomly divided into six groups (*n* = 8). These six groups were injected intraperitoneally once per day with vehicle, 20 mg/kg dbcAMP, 20 mg/kg.

Luteolin, 5 mg/kg FH535, dbcAMP+FH535, or Luteolin+FH535 respectively for 14 days. Tumor diameters were measured every other day with a caliper, and tumor volumes were estimated using the following formula: V (mm^3^) = width^2^ × length/2. Tumors were dissected and fixed in formalin for immunohistochemical analysis. Data are means ± SD of 8 mice/group.

The orthotopic implantation of glioma cells was performed using 5 × 10^4^ GSCs. In brief, cells were injected 2 mm lateral and 0.5 mm anterior to the bregma and 2.5 mm below the skull of 4-week-old athymic nude mice. After 3 days, the mice were randomly divided into four groups and injected intraperitoneally with vehicle, 20 mg/kg dbcAMP+20 mg/kg Luteolin (dL group), 5 mg/kg FH535, or dL+FH535, once per day for 14 days. The mice were monitored daily and sacrificed when neurological symptoms were observed. Their brains were then dissected and fixed in formalin for H&E staining. A Kaplan–Meier survival curve was generated using the GraphPad Prism 6.0 software package (GraphPad Software, La Jolla, CA, USA). The points on the curves indicate glioma-related deaths (*n* = 7 animals for each group; *p* was determined by log-rank analysis).

### Statistical analysis

Statistical differences were evaluated with Student’s *t* test (two-tailed, unequal variance) or ANOVA followed by LSD post hoc test using the SPSS 20 statistical software package (IBM, New York, NY, USA). Statistical significance was defined as *p* values ≤ 0.05.

## Results

### cAMP drives neuronal differentiation in GSCs

**Fig. 1:**
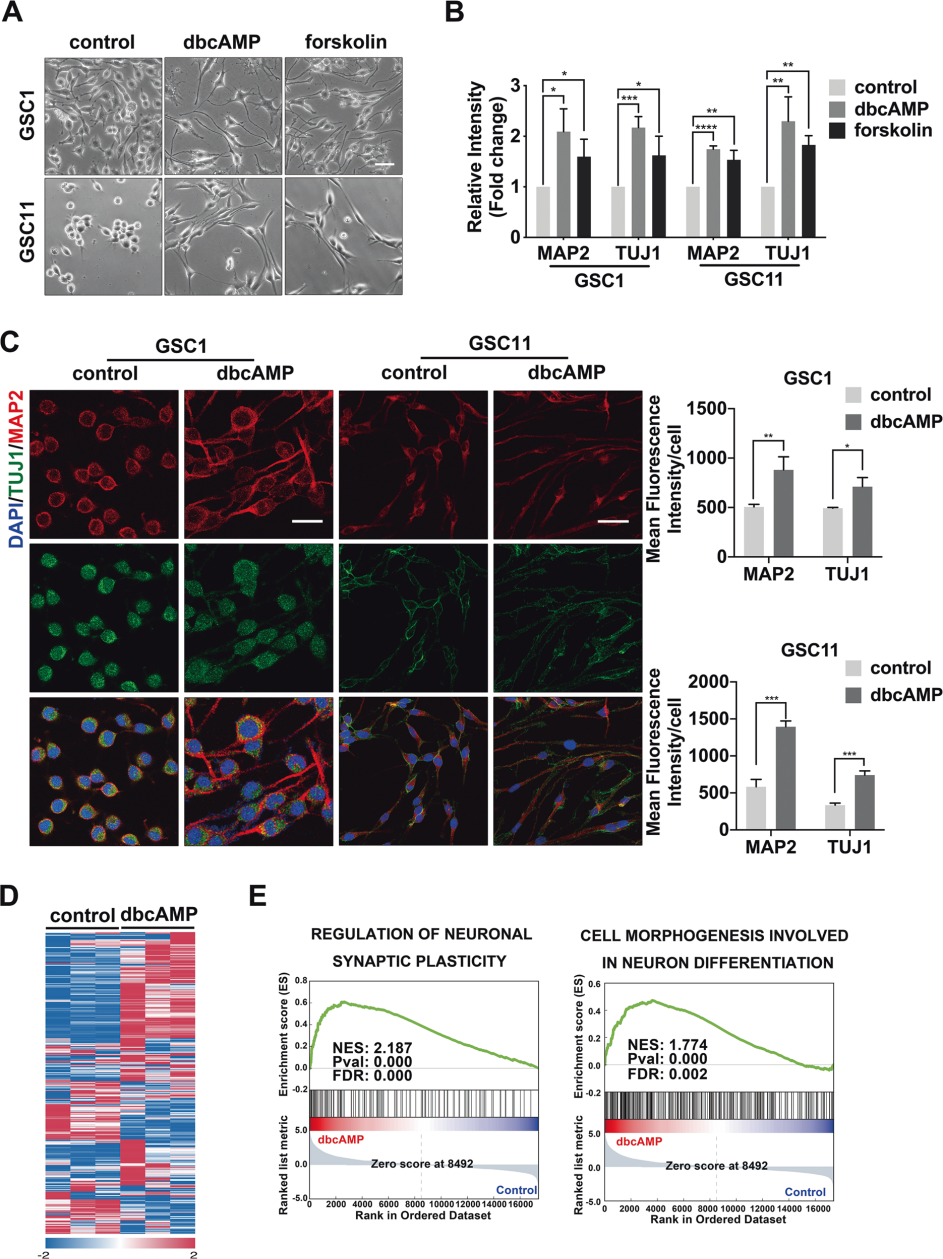
cAMP induces neuronal differentiation in GSCs. **A** Phase-contrast image of GSC1 and GSC11 cells before and after treatment with dbcAMP and forskolin for 72 h (scale bar, 100 μm). **B** Quantification of MAP2 and TUJ1 in GSCs treated with dbcAMP and forskolin by flow cytometry. **C** Immunofluorescence staining of MAP2 (red) and TUJ1 (green) in GSCs before and after incubation with dbcAMP (left) and quantification of fluorescence intensity (right) (scale bar, 50 μm). **D** Heat map of differentially expressed genes between GSCs treated with and without dbcAMP. **E** GSEA analysis using the mRNA sequencing data. (Data are shown as the mean ± SD. N.S., not significant; *, *p* < 0.05; **, *p* < 0.01; ***, *p* < 0.001; ****, *p* < 0.0001).

Previous studies have reported that the activation of cAMP induces differentiation in the established glioma cell lines [33, 34]. To examine the impact of cAMP activation on patient-derived GSCs, we incubated two primary GSC lines (GSC1 and GSC11) with dibutyryl cyclic AMP (dbcAMP), a cAMP analog, and forskolin, an adenylate cyclase activator. We observed that GSCs exhibited morphological changes such as the development of neuron-like elongation after incubation for 72 h (Fig. 1A). Flow cytometry and immunofluorescence staining indicated that GSCs underwent a strong neuronal differentiation evidenced by the increased expression of neuron lineage markers MAP2 and TUJ1 after exposure to cAMP activators (Fig. 1B, C). To further confirm the induction of neuronal differentiation, we performed transcriptome profiling and found that genes related to neuronal differentiation and synaptic plasticity were significantly enriched in dbcAMP-exposed GSCs (Fig. 1D, E).

### Activation of cAMP impairs self-renewal and inhibits proliferation in GSCs

**Fig. 2:**
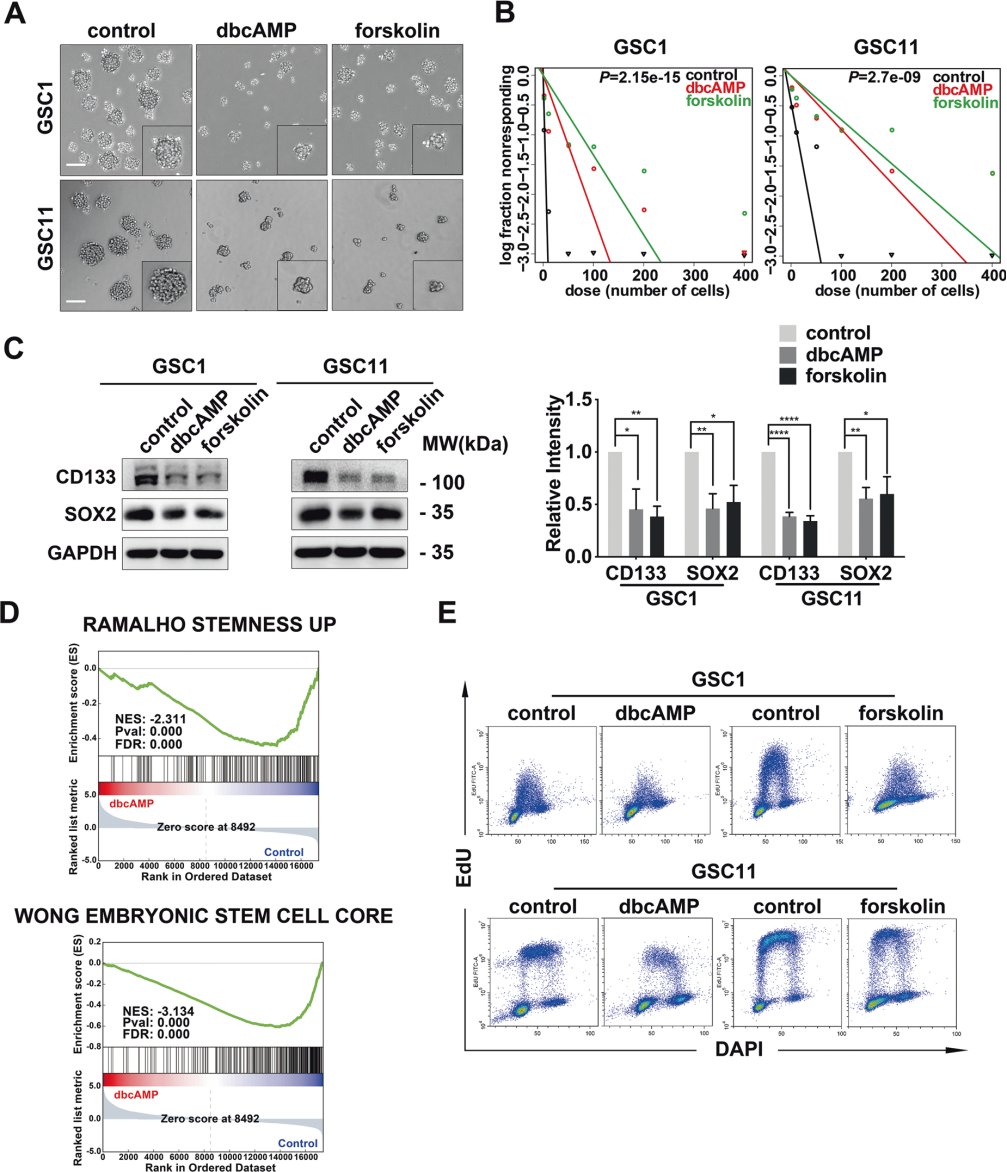
Activation of cAMP compromises self-renewal and represses proliferation in GSCs. **A** Phase-contrast images of neurospheres formed by GSC1 and GSC11 cells after treatment with cAMP activators for 72 h. **B** Limiting dilution assays of GSC1 and GSC11 cells treated with dbcAMP and forskolin. **C** Western blot of CD133 and SOX2 protein in GSCs exposed to dbcAMP and forskolin (left) and quantification of protein bands (right). **D** GSEA analysis using the mRNA sequencing data. **E** Flow cytometry and EdU incorporation DNA histograms of GSCs before and after the treatment with dbcAMP and forskolin. (scale bar, 100 μm; data are shown as the mean ± SD. N.S., not significant; *, *p* < 0.05; **, *p* < 0.01; ***, *p* < 0.001; ****, *p* < 0.0001).

To determine whether the cAMP-induced differentiation is accompanied by the impairment of stemness in GSCs, we initially evaluated the effect of cAMP activation on the neuroshpere formation of GSCs. Quantification analysis demonstrated that the size of neurospheres was reduced by more than 50% when GSCs were treated with dbcAMP or forskolin (Fig. 2A and Supplementary Fig. S2A). We next employed the limiting dilution assay to quantify GSC frequency and self-renewal activity [23]. Both cAMP activators significantly diminished the proportion of self-renewing sphere forming unit (Fig. 2B). Meanwhile, the protein level of neural stem cell markers CD133 and SOX2 were significantly decreased by cAMP activation (Fig. 2C). Gene set enrichment analysis (GSEA) revealed that two stemness representative gene sets, “Stemness up” and “Embryonic stem cell core”, were downregulated by dbcAMP (Fig. 2D). Moreover, exposure of GSCs to dbcAMP or forskolin resulted in a notable decrease in cell proliferation. As shown in Fig. 2E and Supplementary Fig. S2B, the proportion of proliferating cells labeled with EdU was reduced by approximately 50% in GSCs after treatment. Collectively, these results indicated that cAMP activation leads to the impairment of stemness and the repression of proliferation in GSCs.

### cAMP positively regulates β-catenin pathway by stabilizing β-catenin through PKA and GSK-3β

**Fig. 3:**
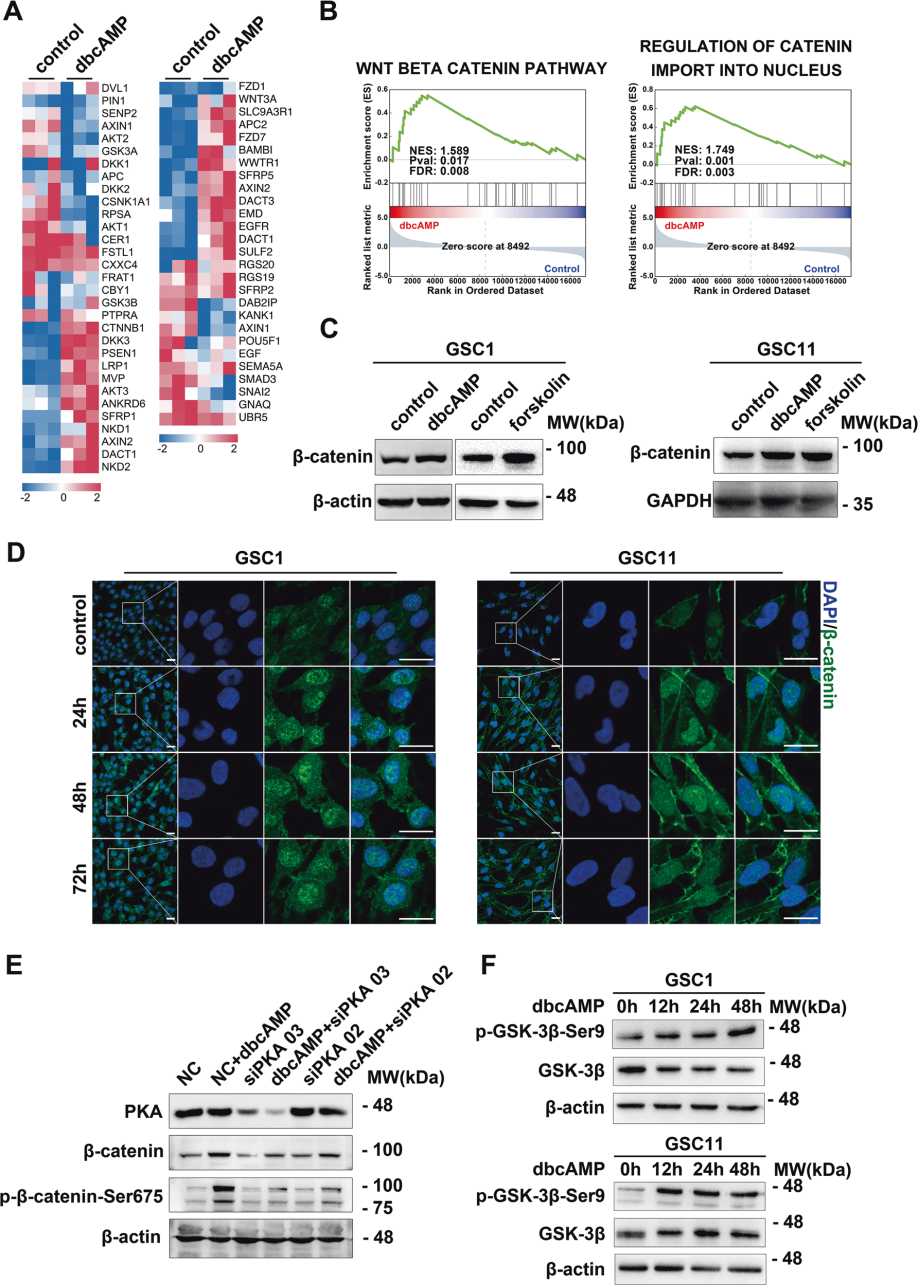
cAMP activates β-catenin signaling by inhibiting destabilization of β-catenin through PKA and GSK-3β. **A** Heat map of DEGs between GSCs treated with and without dbcAMP. **B** GSEA analysis using the mRNA sequencing data. **C** Western blot of β-catenin protein in GSCs incubated with dbcAMP and forskolin. **D** Immunofluorescence staining of β-catenin (green) in GSCs treated with dbcAMP at 0 (control), 24, 48, and 72 h. **E** Western blot of total and phosphorylated β-catenin in GSCs subjected to different treatments. **F** Protein level of phosphorylated GSK-3β at Ser9 and GSK-3β assessed by Western blot in GSCs treated with dbcAMP at 0, 24, 48, and 72 h. (Scale bar, 25 μm).

The mechanisms underlying cAMP-induced neuronal differentiation in GSCs have not been fully elucidated. To investigate pathways involved in the differentiation process, we explored the signaling signatures in GSCs incubated with dbcAMP. We identified that genes closely related to the Wnt/β-catenin pathway and those enhancing nuclear translocation of β-catenin were enriched in dbcAMP-treated GSCs (Fig. 3A, B). The protein level of β-catenin increased in GSCs after incubation with dbcAMP and forskolin (Fig. 3C and Supplementary Fig. S3A). Additionally, the immunofluorescence assay demonstrated that dbcAMP significantly increased the total and nuclear level of β-catenin in GSCs (Fig. 3D and Supplementary Fig. S3B).

PKA as a key downstream effector of cAMP has been found to inhibit the ubiquitination of β-catenin by phosphorylating the molecule at Ser675 [35]. To determine whether PKA is responsible for the stabilization of β-catenin, we transiently knocked down PKA in GSCs incubated with or without cAMP agonist. The result showed that the knock-down of PKA significantly abolished the phosphorylation of β-catenin at Ser675 and led to the decrease in the level of β-catenin (Fig. 3E and Supplementary Fig. S3C). In addition, GSK-3 is a major negative regulator of the Wnt signaling by triggering β-catenin destabilization [36]. It has been reported that increased level of cAMP/PKA pathway activity promotes neuronal survival by inactivating GSK-3β [37]. We then asked whether cAMP agonists could enhance β-catenin signaling through modulating GSK-3β activity. We found that dbcAMP stimulated the inhibitory phosphorylation at Ser9 of GSK-3β in a time-dependent manner (Fig. 3F and Supplementary Fig. S3D). Overall, the evidence above illustrated that cAMP activates β-catenin signaling by stabilizing β-catenin through PKA and GSK-3β.

### Suppression of β-catenin signaling promotes cAMP-induced neuronal differentiation in GSCs

**Fig. 4:**
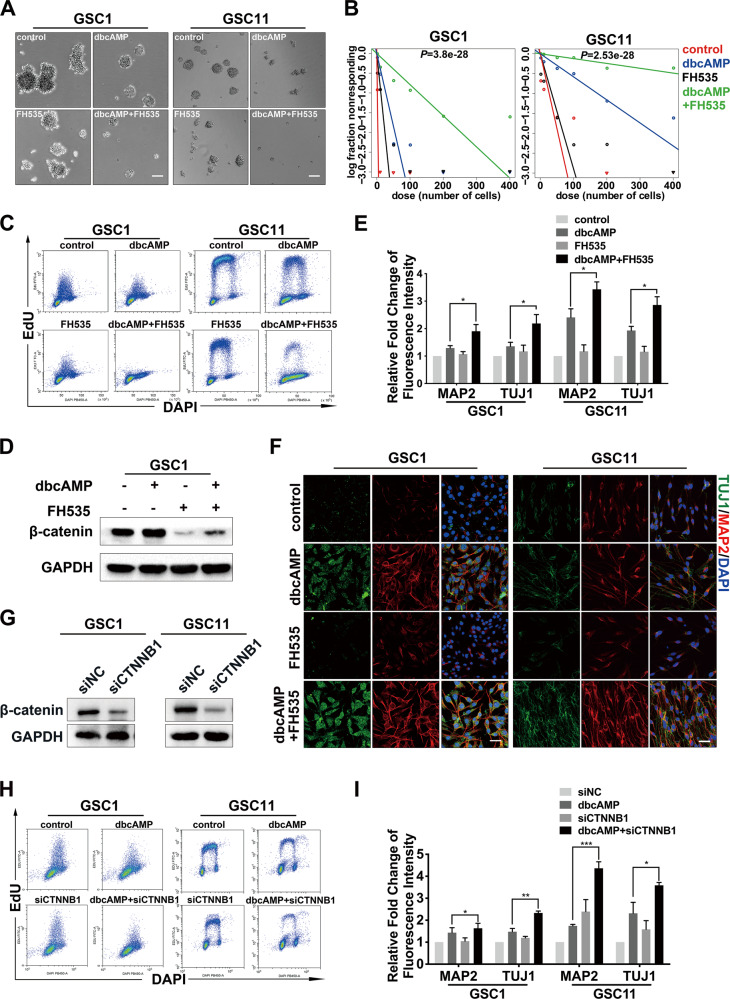
Suppression of β-catenin promotes cAMP-induced differentiation in GSCs. **A** Phase-contrast image of neurospheres formed by GSC1 and GSC11 cells after various treatments for 72 h (scale bar, 100 μm). **B** Limiting dilution assays of GSC1 and GSC11cells treated with dbcAMP, FH535, and the combination of dbcAMP and FH535. **C** Proliferation was evaluated by EdU incorporation analysis in GSCs treated with dbcAMP, FH535, and the combination of the two agents. **D** Western blot of β-catenin in GSC1 treated with dbcAMP and FH535. **E** Quantification by flow cytometry of MAP2 and TUJ1 in GSCs incubated with dbcAMP and FH535. **F** Immunofluorescence staining of MAP2 (red) and TUJ1 (green) in GSCs with different treatments (scale bar, 50 μm). **G** Western blot of β-catenin in GSCs treated with siRNA specific for β-catenin (siCTNNB1). **H** Proliferation evaluated by EdU incorporation analysis of GSCs with β-catenin knock-down. **I** Quantification of MAP2 and TUJ1 in GSCs with β-catenin knock-down by using flow cytometry. (Data are shown as the mean ± SD. N.S., not significant; *, *p* < 0.05; **, *p* < 0.01; ***, *p* < 0.001).

β-catenin has been found to promote differentiation in stem cells, although conflicting reports also exist [38, 39]. To determine the role of β-catenin signaling in the differentiation of GSCs triggered by cAMP, we co-treated GSCs with dbcAMP and a potent inhibitor for β-catenin, FH535. Results from the neurosphere assay showed that dbcAMP combined with FH535 dramatically decreased the size of GSC1 and GSC11 neurospheres compared to monotherapy with either agent (Fig. 4A and Supplementary Fig. S4A). The limiting dilution assay exhibited that β-catenin inhibition cooperated with cAMP activation to repress the self-renewal in GSCs (Fig. 4B). As shown in Fig. 4C and Supplementary Fig. S4B, FH535 significantly enhanced cAMP-induced proliferation arrest in GSCs. Moreover, FH535 prevented the elevation of β-catenin protein level mediated by cAMP activation (Fig. 4D and Supplementary Fig. S4C). FCM demonstrated that the combination treatment dramatically increased the level of MAP2 and TUJ1 in GSCs, compared to the monotherapy with dbcAMP (Fig. 4E). Immunofluorescence staining confirmed that attenuated β-catenin signaling significantly promoted the expression of MAP2 as well as TUJ1 in GSCs after cAMP activation (Fig. 4F and Supplementary Fig. S4D). Similar findings that reduction of β-catenin resulted in decreased proliferation and elevated expression of neuronal markers in dbcAMP-incubated GSCs were confirmed by using specific siRNA (siCTNNB1) (Fig. 4G–I, Supplementary Fig. S4E, S4F). These data suggested that the activation of β-catenin impedes cAMP-induced differentiation in GSCs and suppression of β-catenin reinforces cAMP-induced differentiation therapy in vitro.

### β-catenin inhibition synergizes with cAMP activators to repress glioma growth in vivo

**Fig. 5:**
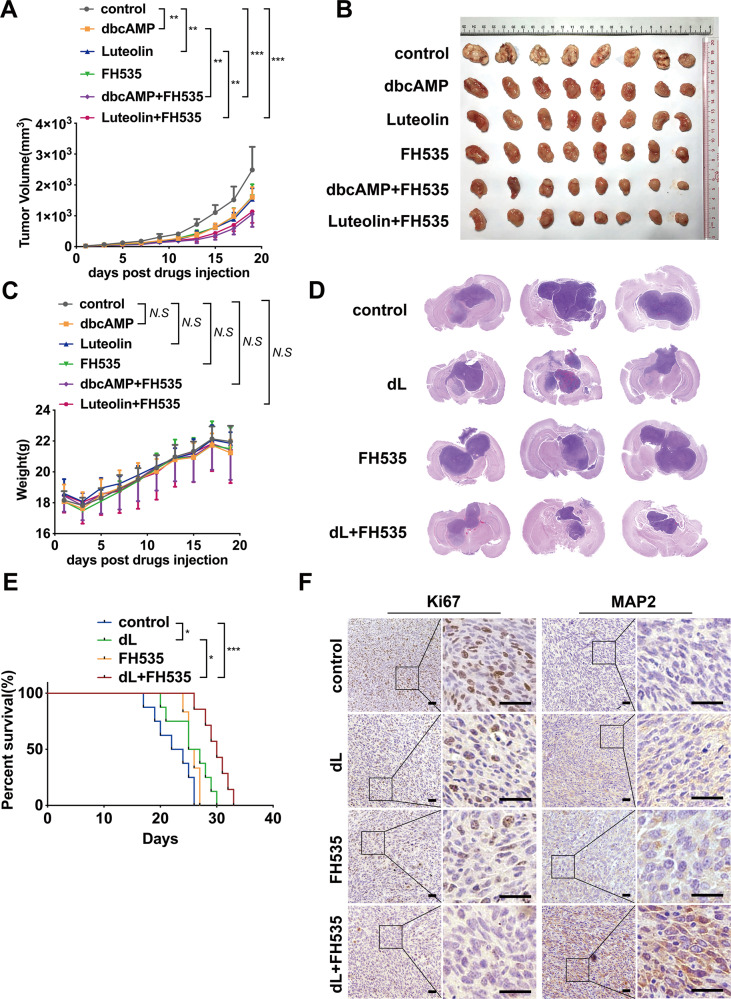
β-catenin inhibitor synergizes with cAMP agonists in GSC xenograft models. **A** Tumor growth curve of subcutaneous mouse model treated with cAMP activators and FH535. **B** Representative photos of subcutaneous tumor specimens in different groups. **C** Changes of the body weights after treatment in various groups. **D** Representative images of mouse brain specimens from orthotopic models treated with vehicle, dbcAMP+Luteolin (dL), FH535, dL+FH535. **E** Kaplan–Meier curve showing survival of mice bearing GSCs treated with cAMP activators, FH535 and the combination. **F** H&E staining of tumors derived from GSCs subjected to different treatments. Tumor tissues were stained with antibodies against Ki-67 and MAP2. (Scale bar, 25 μm; Data are shown as the mean ± SD. N.S., not significant; *, *p* < 0.05; **, *p* < 0.01; ***, *p* < 0.001).

To explore whether the cooperative effect of β-catenin deactivation and cAMP agonists occurs in vivo, we implanted GSCs subcutaneously in immunocompromised mice. After the establishment of tumors, mice were treated with vehicle, cAMP activators (dbcAMP or Luteolin), FH535, and combined cAMP activators with FH535, respectively. Monotherapy with the agents mentioned above mildly retarded the glioma growth whereas a more notable decrease in tumor size was observed in mice administered with cAMP activators and FH535 in combination (Fig. 5A, B). The body weight was comparable in each group, suggesting that the drug combination had insignificant toxicity (Fig. 5C). We validated these results by using an orthotopically xenografted model. The combined cAMP activators and FH535 significantly inhibited the glioma progression and extended the survival of the animals, compared to monotherapy with either cAMP activators or FH535 (Fig. 5D, E). Analysis of tumor sections identified more evidence of decreased tumor proliferation (Ki-67) and elevated expression of neuronal maker MAP2 in the group with combined treatment (Fig. 5F and Supplementary Fig. S5). All the results described above indicated that the β-catenin inhibitor synergizes with cAMP activators to suppress glioma growth and trigger neuronal differentiation in vivo.

### APELA and CARD16 are downstream effectors of β-catenin that contribute to the inhibited differentiation in cAMP-treated GSCs

**Fig. 6:**
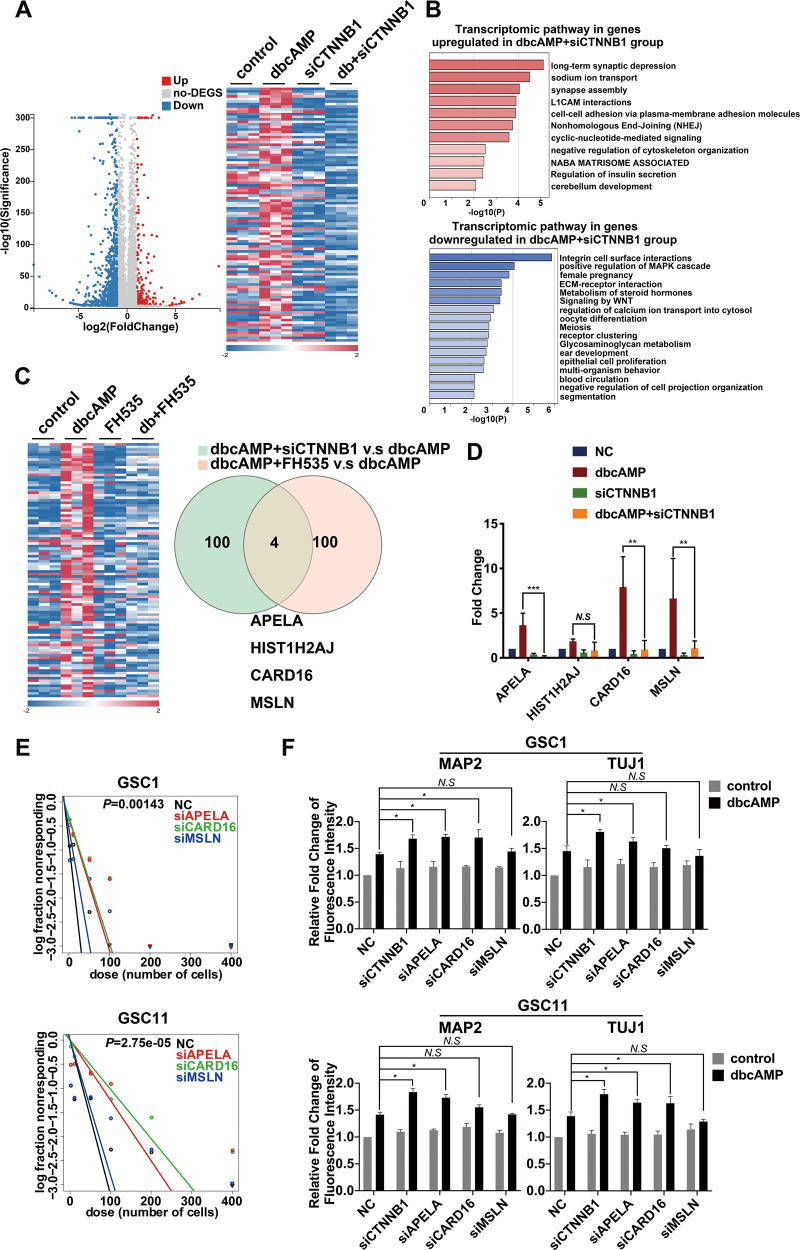
Identification of β-catenin downstream genes that contribute to the negative regulation of cAMP-induced differentiation in GSCs. **A** Volcano plot and heat map show the distribution of DEGs in dbcAMP-treated GSCs before and after the knock-down of β-catenin. **B** Function and pathway enrichment of DEGs in dbcAMP-treated GSCs before and after the knock-down of β-catenin. **C** Heat map and Venn diagram demonstrating the overlap of top downregulated DEGs in the two gene sets. **D** Validation of the expression of four DEGs by using qRT-PCR. **E** Limiting dilution assays of GSC1 and GSC11 cells after treatment with specific siRNA targeting APELA, CARD16 and MSLN. **F** Quantification of MAP2 and TUJ1 in GSCs treated with the combination of dbcAMP and knock-down of CTNNB1, APELA, CARD16 or MSLN, by using flow cytometry. (db, dbcAMP; Data are shown as the mean ± SD. N.S., not significant; *, *p* < 0.05; **, *p* < 0.01; ***, *p* < 0.001).

To understand the biological function of genes downstream of β-catenin in cAMP-treated GSCs, we firstly analyzed the gene expression profiles of GSCs treated with dbcAMP in the presence of silencing RNA against β-catenin. A total of 749 differential expression genes (DEGs; 116 upregulated and 633 downregulated) were found after β-catenin knock-down in GSCs treated with dbcAMP, compared to those incubated with dbcAMP alone (Fig. 6A). The gene annotation and enrichment analysis demonstrated that the DEGs were concentrated in biological processes closely related to differentiation and development such as oocyte differentiation, synapse assembly, and cerebellum development (Fig. 6B). To identify downstream genes of β-catenin that are potentially responsible for the negative regulation of differentiation, we also performed the transcription profiling of GSCs treated with dbcAMP as well as those treated with the combination of dbcAMP and FH535 and compared the DEGs with those obtained from the above β-catenin knock-down experiment. Among the top 100 top downregulated DEGs in the two gene sets, four genes (APELA, HIST1H2AJ, CARD16, and MSLN) were found to overlap (Fig. 6C). qRT-PCR confirmed that the transcript level of 3 genes (APELA, CARD16, and MSLN) significantly differed between GSCs treated with dbcAMP alone and those with combined dbcAMP and β-catenin knock-down (Fig. 6D). To determine the role of these 3 genes in the maintenance of stemness in GSCs, we treated GSCs with specific siRNA for these genes. The limiting dilution assay demonstrated that knock-down of APELA and CARD16 but not MSLN significantly decreased the frequency of neurosphere-forming potential in both GSC1 and GSC11 cells (Fig. 6E). Moreover, we investigated the influence of these three genes on differentiation. FCM demonstrated that knock-down of APELA combined with dbcAMP significantly increased levels of MAP2 and TUJ1 in both GSC1 and GSC11 cell lines, compared to dbcAMP incubation alone. Downregulation of CARD16 synergized with dbcAMP to evidently promote the expression of MAP2 in GSC1 and TUJ1 in GSC11 cells respectively. No significant difference in the level of MAP2 and TUJ1 was observed in GSCs after the knock-down of MSLN (Fig. 6F). Collectively, these results implicated that APELA and CARD16 are upregulated by β-catenin and participate in the inhibition of cAMP-induced differentiation.

## Discussion

**Fig. 7:**
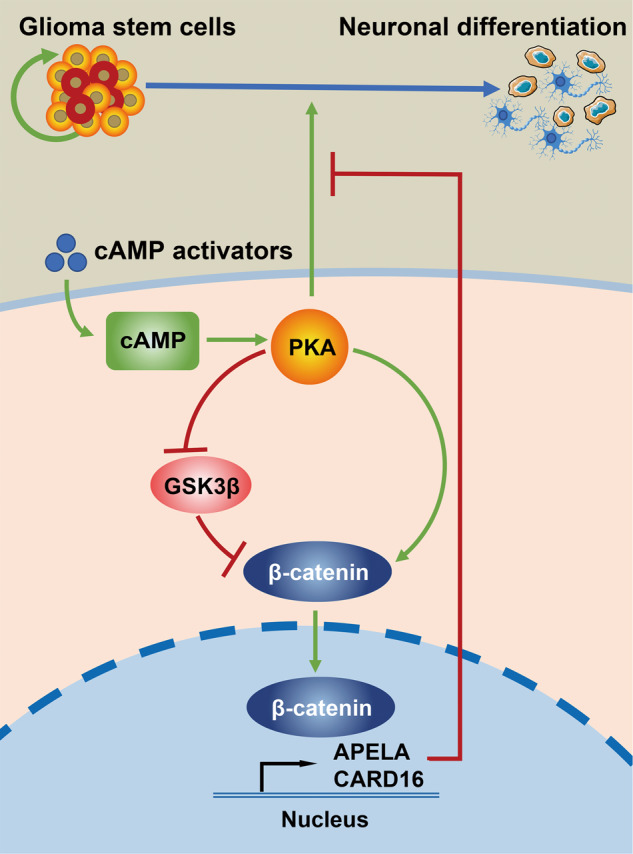
The schematic diagram shows the proposed mechanism that cAMP activates β-catenin through PKA and GSK3β, and afterwards promotes transcription of the downstream genes APELA and CARD16, which eventually prevent neuronal differentiation in GSCs.

GBMs are the most common malignant primary brain cancers in adults with devastating outcomes. Most of the current treatments mainly focus on the glioma bulk mass and fail to be curative [40]. High cellular and genetic heterogeneity of GBMs account for the resistance to the standard-of-care therapy [41]. A small population of GSCs resident in the tumor bulk are responsible for the refractory features by self-renewal and unlimited asymmetric division [42]. Resembling normal stem cells, GSCs may respond to differentiation cues but fail to fully lose their stemness. Deciphering intrinsic pathways that inhibit the differentiation in GSCs and identifying strategies that effectively targeting these signals are promising for glioma treatment. In the current study, we demonstrated that the cAMP activators drive GSCs to differentiate towards neurons. We identified a negative differentiation feedback mechanism, by which cAMP triggers the activation of β-catenin and subsequently increases transcription of the downstream genes APELA and CARD16, which negatively impact neuronal differentiation in GSCs (Fig. 7). Notably, inhibition of β-catenin signaling effectively enhances cAMP-induced differentiation.

The role of cAMP in regulating differentiation in gliomas has been established for more than four decades. The early observation that cAMP promotes morphological and biochemical changes of glioma cells towards mature glia elicited the interest in the feasible application of differentiation therapy in gliomas [43, 44]. Subsequent studies unveiled that PKA is responsible for the cAMP-induced differentiation in gliomas by phosphorylating CREB to initiate the transcription of downstream genes [13, 45]. In fact, a complicated regulatory network is involved in the differentiation process induced by cAMP. Mai and colleagues identified miR-1275 as a repressor of glial induction of glioma cells. cAMP signaling suppresses the expression of miR-1275 and triggers differentiation through epigenetic modification [46]. In this study, we identified the cross-talk between cAMP/PKA and Wnt/β-catenin signaling in GSCs. The administration of cAMP agonists promotes the activation of β-catenin and subsequent transcription of its downstream genes. Mechanistically, cAMP signaling abolishes the ubiquitin-dependent degradation of β-catenin through direct phosphorylation of the molecule by PKA and the inhibition of GSK-3β activity.

Wnt/β-catenin signaling is a critical mediator that regulates embryonic development and tissue homeostasis. β-catenin has been found to be essential for cell fate determination and differentiation. Knockout of β-catenin is lethal to mouse embryonic stem cells (mESC) due to the failure of mESC to develop the endodermal and mesodermal germ layers [47]. In addition, β-catenin modulates mESC differentiation process by maintaining the genomic stability through epigenetic regulatory mechanisms [48]. Loss of β-catenin leads to apoptosis in mESC at the onset of differentiation [49]. The role of β-catenin in sustaining stem cell phenotype in cancers is under debate. The majority of evidence support that β-catenin promotes the maintenance of the CSC pool and inhibits differentiation due to its strong oncogenic capacity; however, several studies draw the contrary conclusions. Chen and colleagues demonstrated that forced activation of β-catenin signaling in embryonal rhabdomyosarcoma leads to compromised self-renewal by inducing myogenic differentiation of CSCs [50]. Similarly, nuclear accumulation of β-catenin promotes expression of markers of melanocyte differentiation in melanomas and results in the inhibited proliferation in vitro and diminished tumor size in vivo [51]. In this study, we initially observed the activation of β-catenin by cAMP agonists in the differentiation process of GSCs. To determine the role of cAMP-triggered β-catenin activation, we repressed β-catenin signaling by pharmacological inhibitor and siRNA. We found in vitro and in vivo that β-catenin signaling functions as a suppressor, rather than an activator of cAMP-induced differentiation in GSCs. The difference in the cell type, genetic background as well as the tumor microenvironment may account for the conflicting reports on the role of β-catenin in the differentiation of cancer stem cells.

We demonstrated that APELA and CARD16 are potential downstream genes of β-catenin, which account for the inhibition of cAMP-induced differentiation in GSCs. Apelin early ligand A (APELA), also called Toddler or ELABELA, is a peptide hormone that binds the G protein-coupled receptor apelin receptor (APLNR) and acts as a critical regulator of gastrulation and cardiovascular development in zebrafish [52, 53]. In human pluripotent embryonic stem cells (hESCs), APELA potentiates PI3K/AKT/mTORC1 signaling through an unknown cell-surface receptor other than APLNR and is required for the maintenance of self-renewal of hESCs by promoting cell-cycle progression and blocking stress-induced apoptosis [54]. Ganguly and colleagues found that APELA is over-expressed in GSCs and localizes in the putative stem cell niche in GBM tissues. Increased APELA transcription is associated with an increased histological grade of gliomas and poor prognosis of patients with GBM [55]. In the current study, we revealed that APELA supports the self-renewal of GSCs and negatively regulates cAMP-induced neuronal differentiation. APELA inhibits differentiation probably by suppressing the activity of adenylate cyclase that catalyzes the conversion of ATP to cAMP [56].

The caspase recruitment domain (CARD) is a member of death domain (DD) superfamily that modulates protein-protein interaction. CARD has been found in a diversity of adaptor proteins and regulates crucial signaling pathways involved in caspase activation, inflammasome assembly and innate immune response [57]. Among more than 30 CARD-containing proteins in human, CARD16 is a small molecule that consists of 97 amino acids with a solitary CARD motif, which has a 92% sequence identity to the prodomain of caspase-1 [58]. During the inflammatory response, CARD16 acts as a decoy to disrupt RIP2 binding to caspase-1 via CARD-CARD interaction, leading to the blockade of caspase-1 activity and the stimulation of NF-κB [59]. Our prior study demonstrated that a 6-gene signature including CARD16 features the tumor immune microenvironment and predicts the prognosis of IDH wild-type gliomas [60]. Herein, we demonstrated that CARD16 participates in the stemness maintenance of GSCs and the inhibition of cAMP-induced differentiation. To our knowledge, the role of CARD16 in cancer stem cells has not been reported previously. The mechanisms underlying the negative influence of CARD16 on differentiation currently remain unknown and deserve further investigation.

In conclusion, we identify a negative feedback mechanism in differentiation therapy for GSCs. cAMP agonists induce neuronal differentiation and simultaneously inhibit the degradation of β-catenin through PKA and GSK3β. The activated β-catenin translocates into the nucleus and promotes the transcription of APELA and CARD16, facilitating the maintenance of the stemness and compromise differentiation in GSCs. Suppression of β-catenin signaling dramatically improves the efficacy of differentiation therapy both in vitro and in vivo. Our findings enhance the understanding of the interaction of signaling pathways in cAMP-induced differentiation and provide attractive targets for the reinforcement of differentiation therapy for GBM.

## Supplementary information

Figure S1

Figure S2

Figure S3

Figure S4

Figure S5

Supplementary figure legends

Reproducibility checklist

Original Data of WB

## Data Availability

The key raw data have been uploaded onto the Research Data Deposit public platform (www.researchdata.org.cn), with the approval RDD number of RDDB2021173387.
