# Supplementary figures and images for: Disruption of β-catenin-mediated negative feedback reinforces cAMP-induced neuronal differentiation in glioma stem cells
Source: Cell Death Dis. 2022 May 24;13(5):493. doi: 10.1038/s41419-022-04957-9 (PMC9130142; doi:10.1038/s41419-022-04957-9)

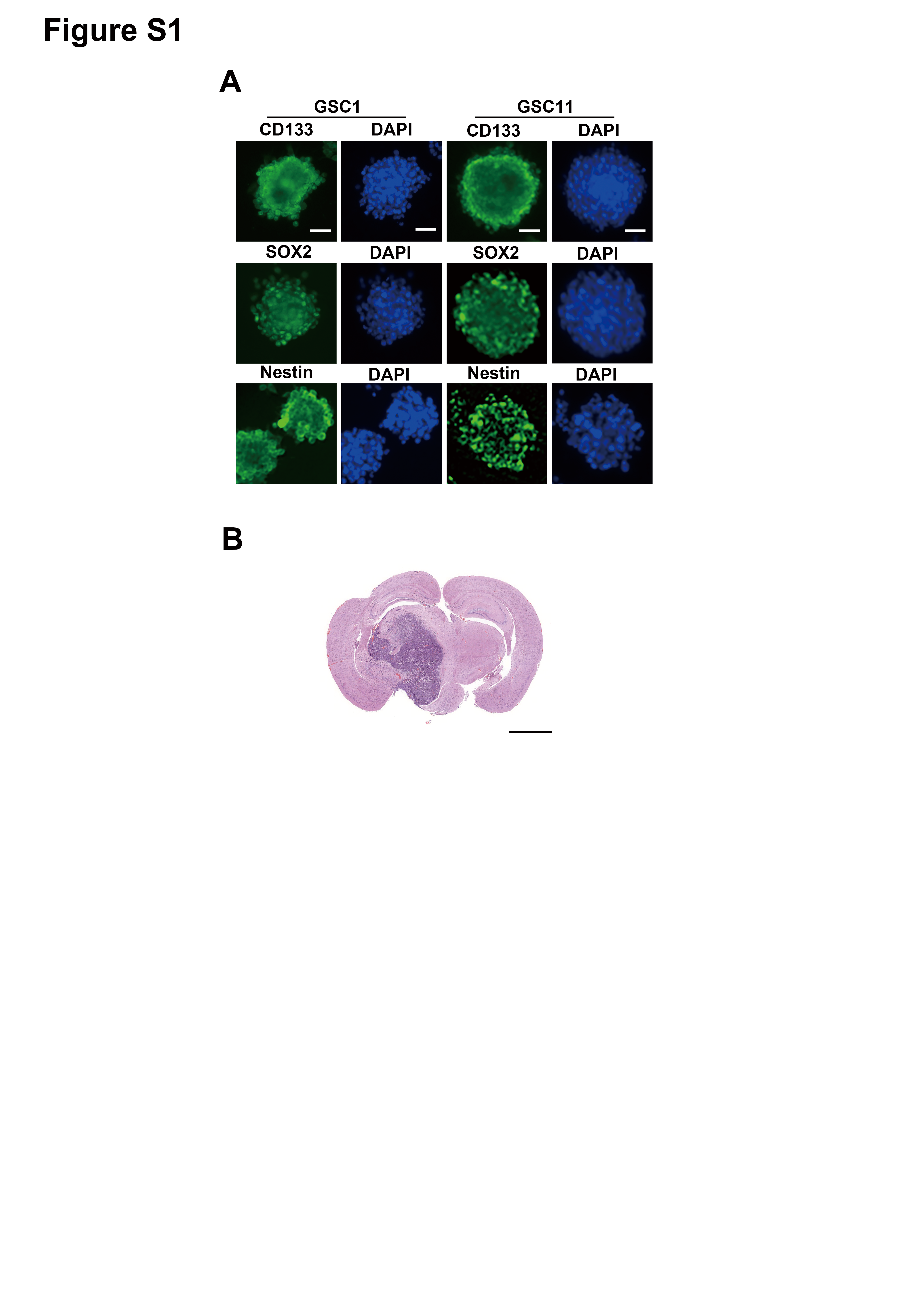

Supplement: Supplementary file 1 — Figure S1 [file 41419_2022_4957_MOESM1_ESM.png]

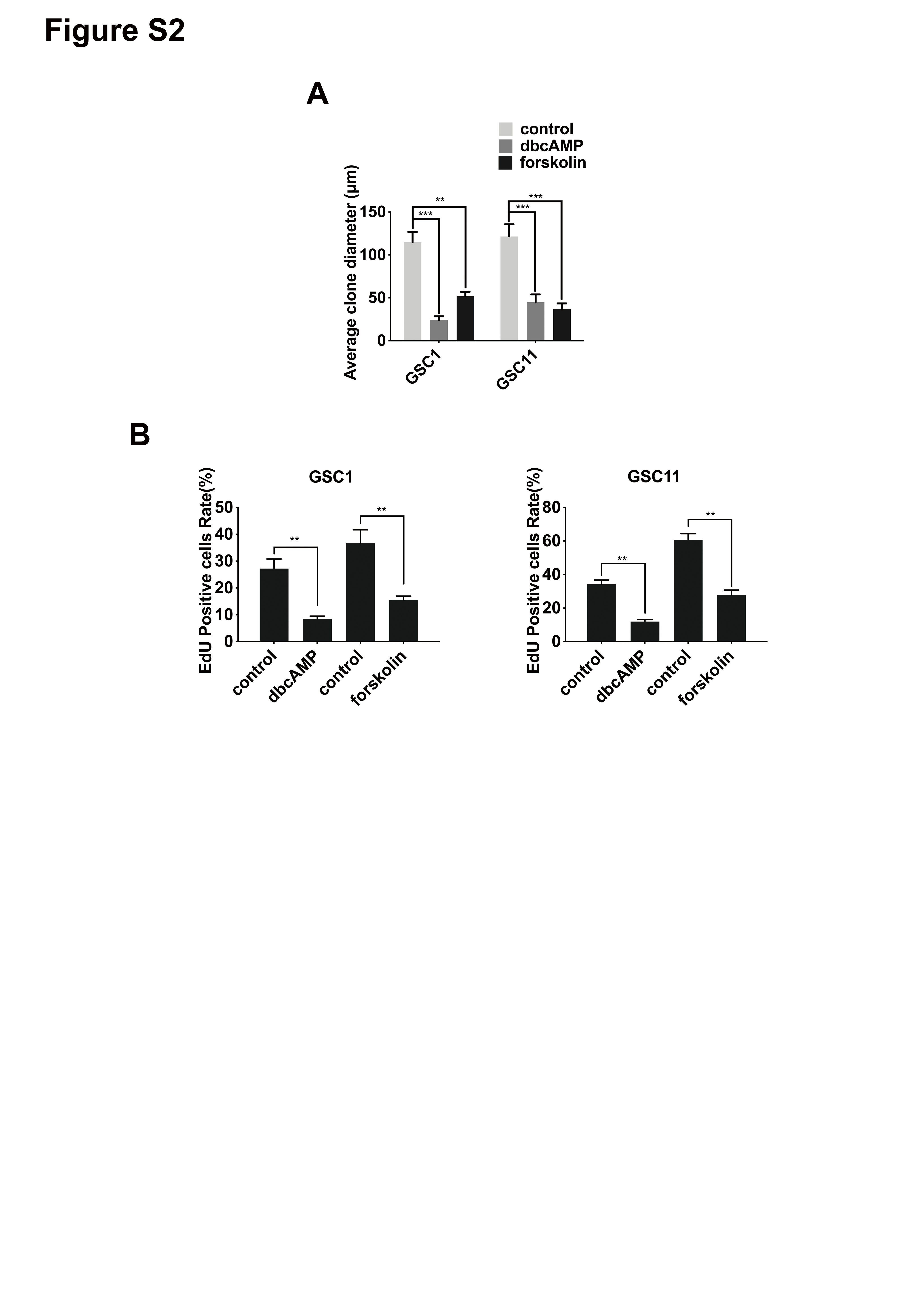

Supplement: Supplementary file 2 — Figure S2 [file 41419_2022_4957_MOESM2_ESM.png]

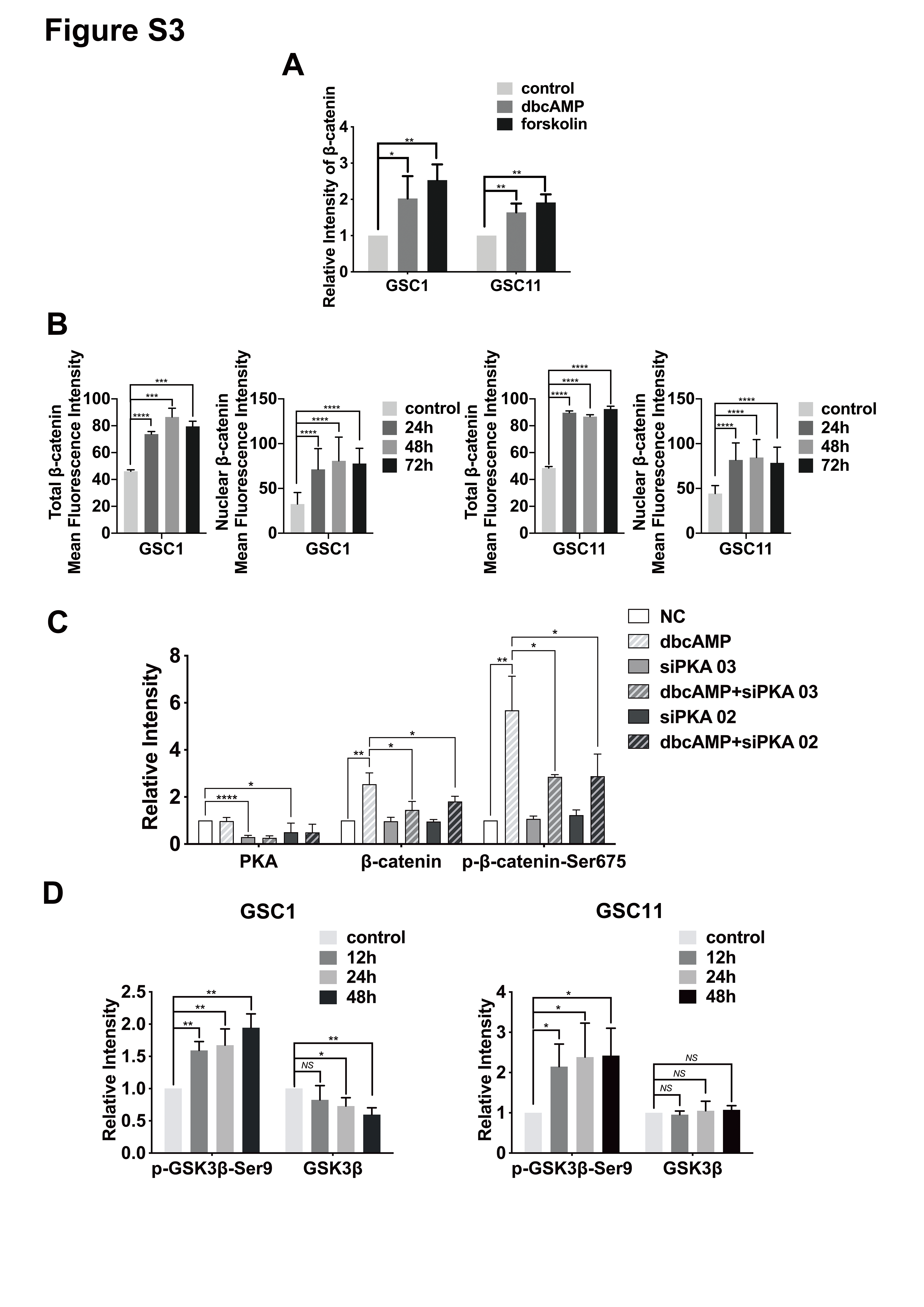

Supplement: Supplementary file 3 — Figure S3 [file 41419_2022_4957_MOESM3_ESM.png]

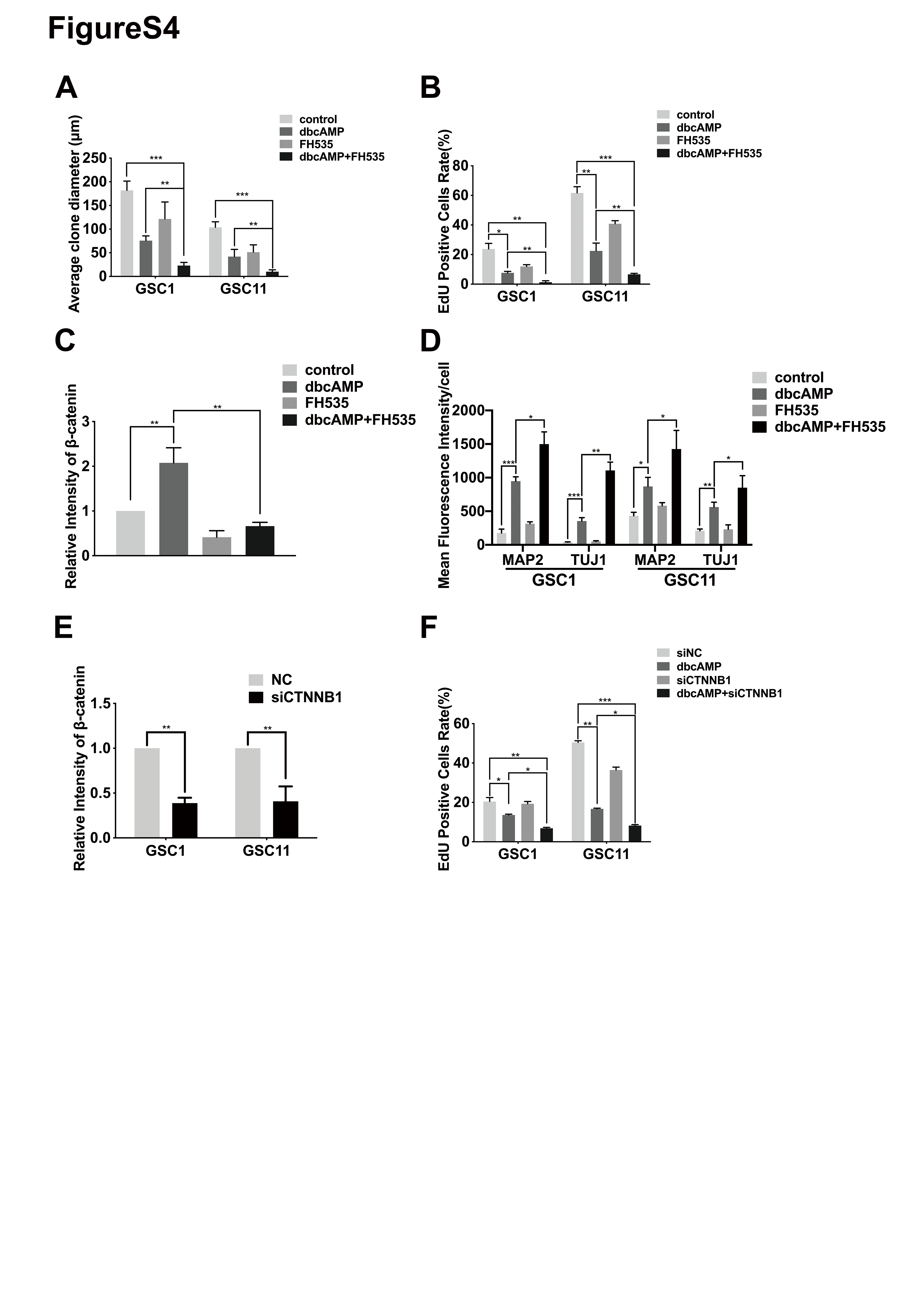

Supplement: Supplementary file 4 — Figure S4 [file 41419_2022_4957_MOESM4_ESM.png]

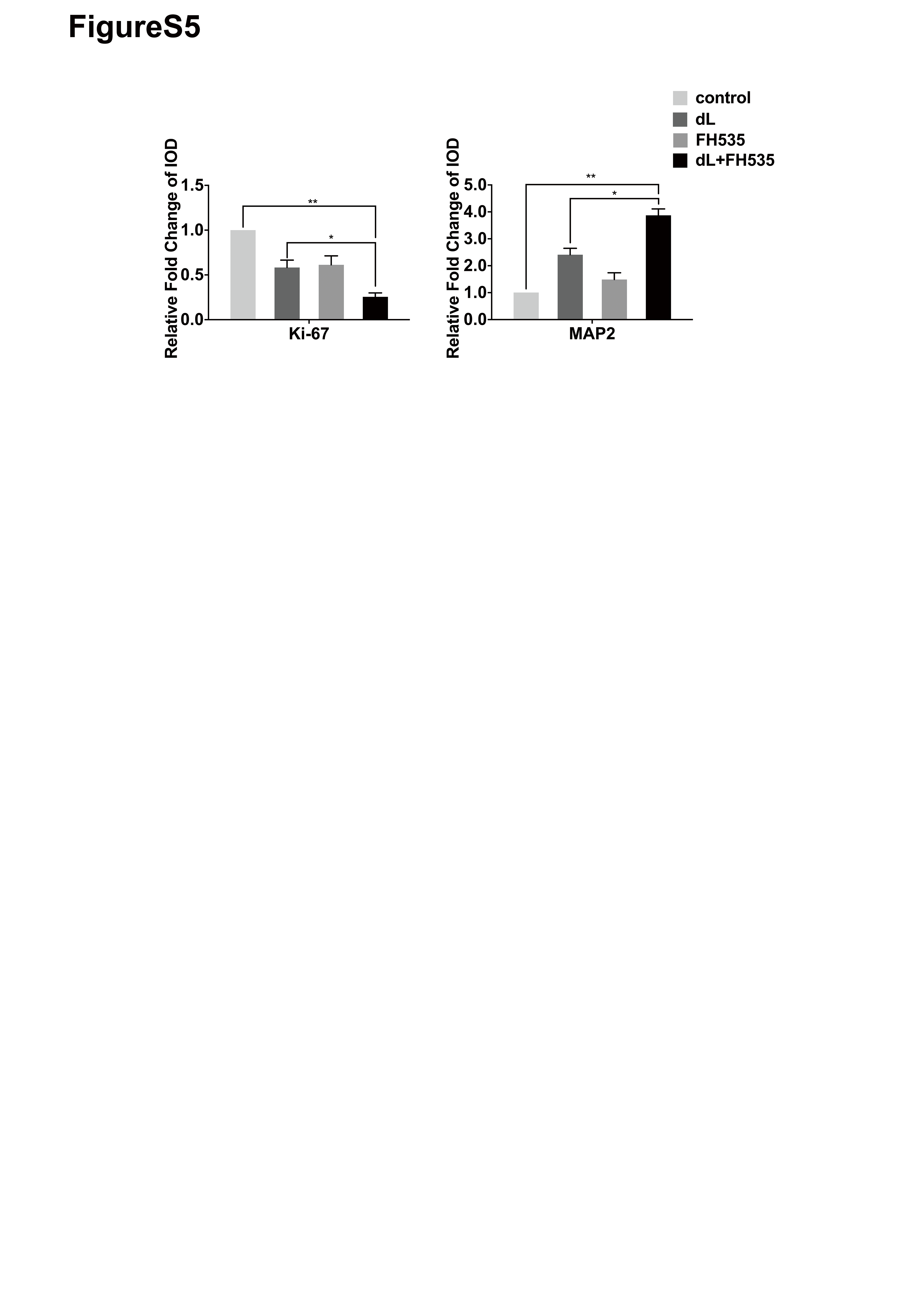

Supplement: Supplementary file 5 — Figure S5 [file 41419_2022_4957_MOESM5_ESM.png]
