# Supplementary material for: Disruption of β-catenin-mediated negative feedback reinforces cAMP-induced neuronal differentiation in glioma stem cells
Source: Cell Death Dis. 2022 May 24;13(5):493. doi: 10.1038/s41419-022-04957-9 (PMC9130142; doi:10.1038/s41419-022-04957-9)
Supplement: Supplementary file 6 — Supplementary figure legends [file 41419_2022_4957_MOESM6_ESM.docx]

**Supplementary figure legends**

**Supplementary Fig. 1 Characterization of GSC cell lines.**

(A) Immunofluorescence staining of CD133, SOX2 and Nestin for GSC1 and GSC11 cells. (B) The representative image of a mouse brain specimen with glioma initiated by GSC1 cells.

**Supplementary Fig. 2**

(A) Quantification of the size of neurospheres in GSCs treated with dbcAMP or forskolin. (B) Analysis of the proportion of EdU (+) GSCs after different treatments. (Data are shown as the mean ± SD. N.S., not significant; *, *p* < 0.05; **, *p* < 0.01; ***, *p* < 0.001)

**Supplementary Fig. 3**

(A) Densitometric quantification of protein bands for β-catenin in GSCs treated with dbcAMP and forskolin. (B) Quantification of immunofluorescence of total and nuclear level of β-catenin in GSCs treated with dbcAMP. (C) Densitometric quantification of protein bands for phosphorylated β-catenin at Ser 675 in GSCs subjected to various treatments. (D) Densitometric quantification of protein bands for phosphorylated GSK-3β at Ser9 and GSK-3β in GSCs treated with dbcAMP. (Data are shown as the mean ± SD. N.S., not significant; *, *p* < 0.05; **, *p* < 0.01; ***, *p* < 0.001; ****, *p* < 0.0001)

**Supplementary Fig. 4**

(A) Quantification of the size of neurospheres in GSCs after different treatments. (B) Analysis of the proportion of EdU (+) GSCs after exposure to different treatments. (C) Densitometric quantification of protein bands for β-catenin in GSC1 treated with dbcAMP and FH535. (D) Quantification of immunofluorescence of MAP2 and TUJ1 in GSCs with different treatments. (E) Densitometric quantification of protein bands for β-catenin in GSCs treated with siRNA specific for β-catenin. (F) Quantification of the fraction of EdU (+) cells in GSCs with different treatments. (Data are shown as the mean ± SD. N.S., not significant; *, *p* < 0.05; **, *p* < 0.01; ***, *p* < 0.001)

**Supplementary Fig. 5**

Quantification of immunohistochemical staining for Ki-67 and MAP2 in different tumor sections. (Data are shown as the mean ± SD. N.S., not significant; *, *p* < 0.05; **, *p* < 0.01)
