# Supplementary material for: Disruption of β-catenin-mediated negative feedback reinforces cAMP-induced neuronal differentiation in glioma stem cells
Source: Cell Death Dis. 2022 May 24;13(5):493. doi: 10.1038/s41419-022-04957-9 (PMC9130142; doi:10.1038/s41419-022-04957-9)

Fig.2C

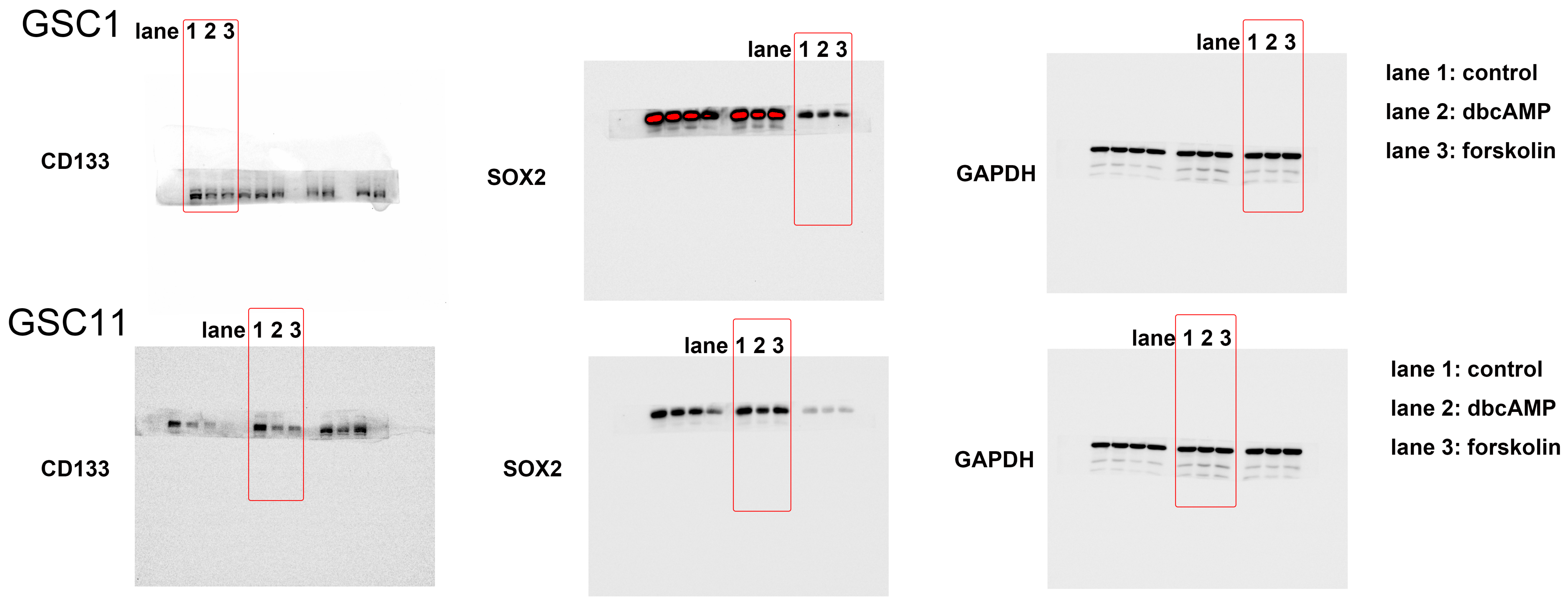
GSC1
lane 1 2 3
CD133
lane 1 2 3
SOX2
lane 1 2 3
GAPDH
lane 1: control
lane 2: dbcAMP
lane 3: forskolin
GSC11
lane 1 2 3
CD133
lane 1 2 3
SOX2
lane 1 2 3
GAPDH
lane 1: control
lane 2: dbcAMP
lane 3: forskolin

## Fig.3C

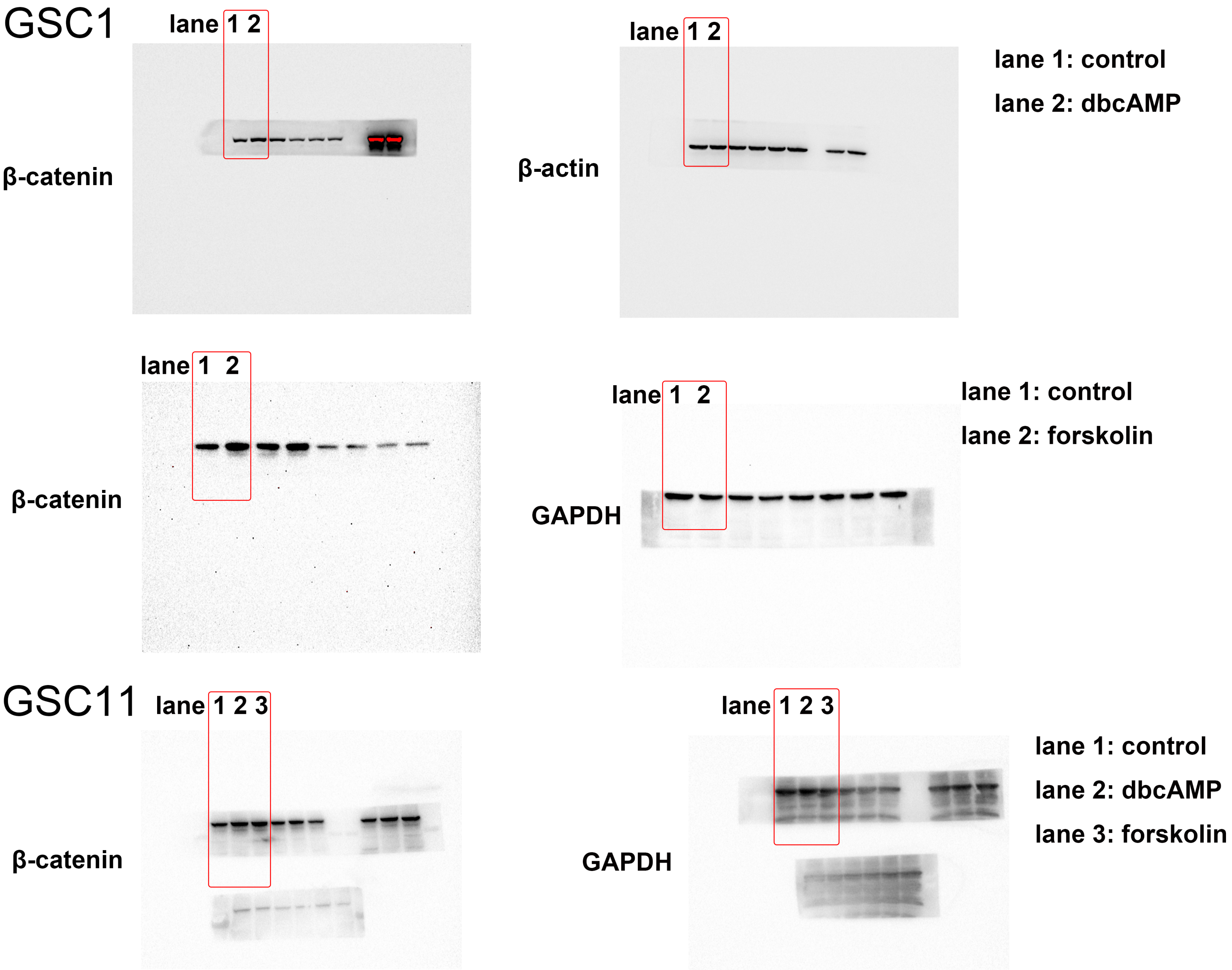
GSC1
lane 1 2
β-catenin
lane 1 2
β-actin
lane 1: control
lane 2: dbcAMP
lane 1 2
β-catenin
lane 1 2
GAPDH
lane 1: control
lane 2: forskolin
GSC11
lane 1 2 3
β-catenin
lane 1 2 3
GAPDH
lane 1: control
lane 2: dbcAMP
lane 3: forskolin

## Fig.3E

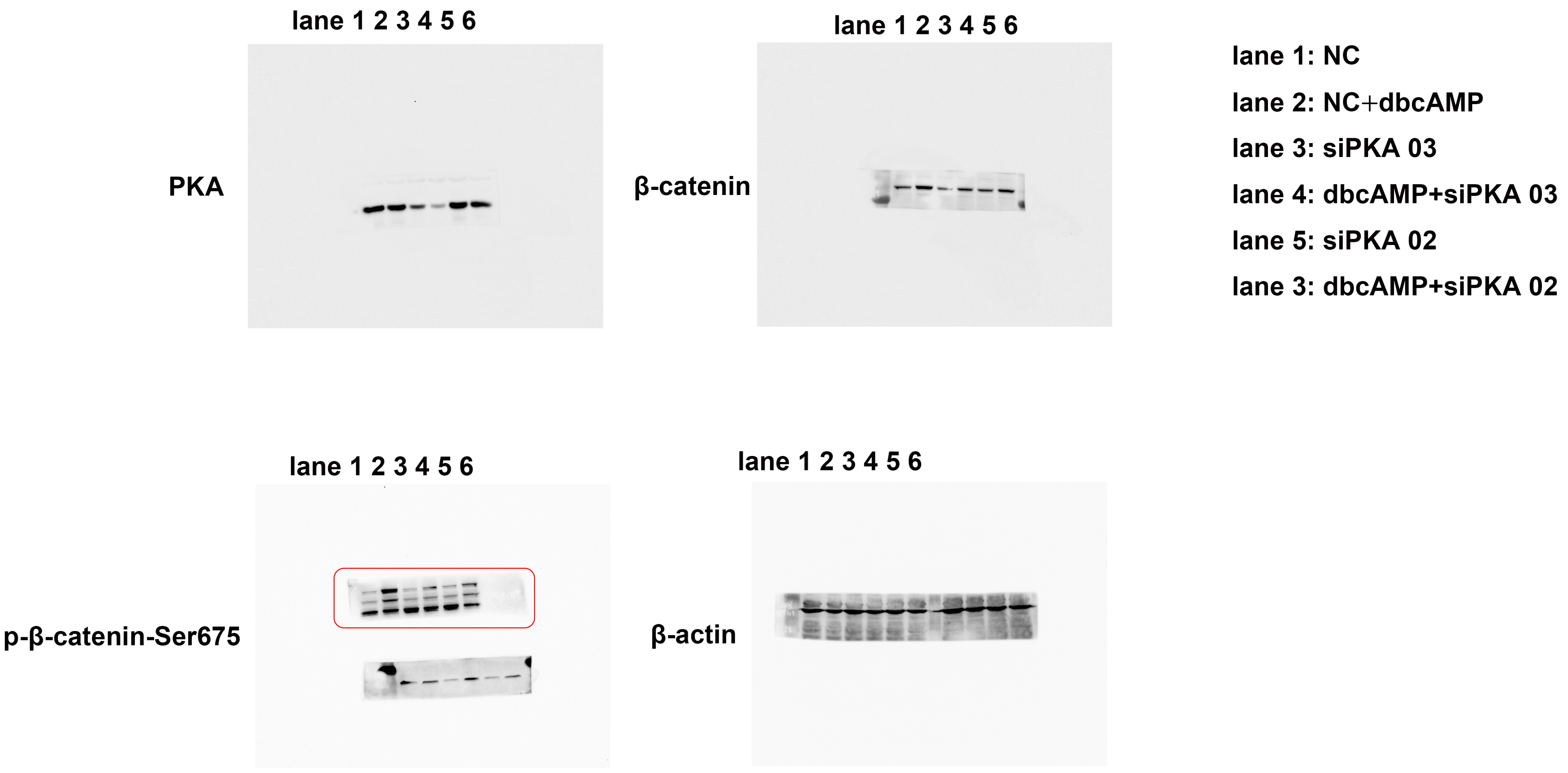
lane 1 2 3 4 5 6
PKA
lane 1 2 3 4 5 6
β-catenin
lane 1: NC
lane 2: NC+dbcAMP
lane 3: siPKA 03
lane 4: dbcAMP+siPKA 03
lane 5: siPKA 02
lane 3: dbcAMP+siPKA 02
lane 1 2 3 4 5 6
p-β-catenin-Ser675
lane 1 2 3 4 5 6
β-actin

Fig.3F

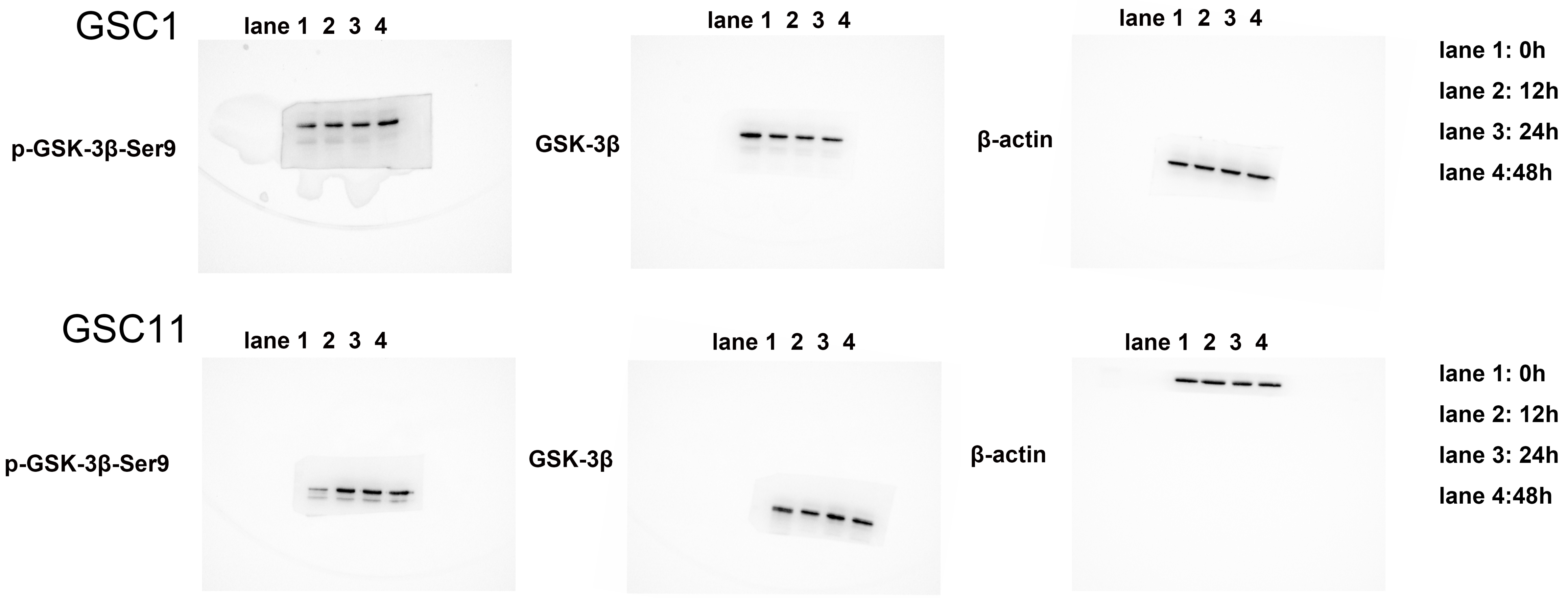
GSC1
lane 1 2 3 4
p-GSK-3β-Ser9
lane 1 2 3 4
GSK-3β
lane 1 2 3 4
β-actin
lane 1: 0h
lane 2: 12h
lane 3: 24h
lane 4:48h
GSC11
lane 1 2 3 4
p-GSK-3β-Ser9
lane 1 2 3 4
GSK-3β
lane 1 2 3 4
β-actin
lane 1: 0h
lane 2: 12h
lane 3: 24h
lane 4:48h

Fig.4D

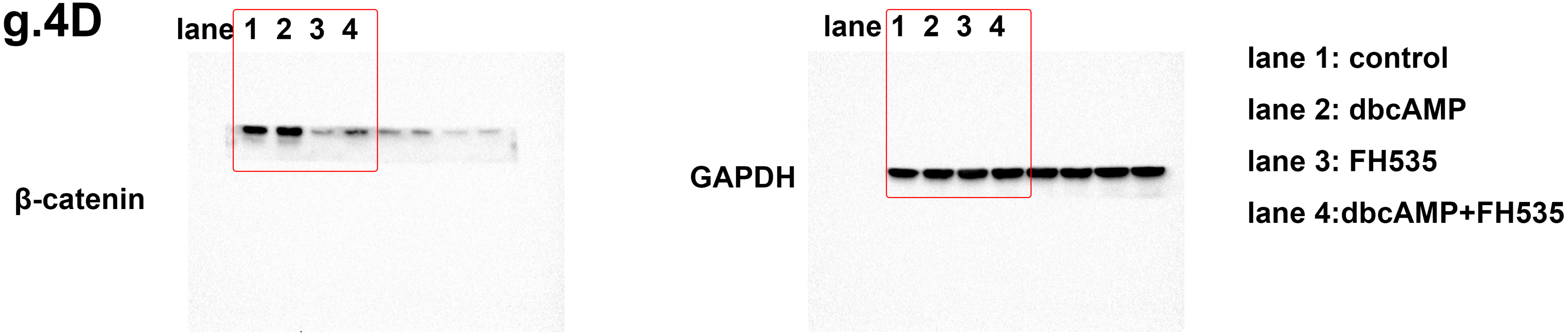
lane 1 2 3 4
β-catenin
lane 1 2 3 4
GAPDH
lane 1: control
lane 2: dbcAMP
lane 3: FH535
lane 4:dbcAMP+FH535

# Fig.4G

GSC1

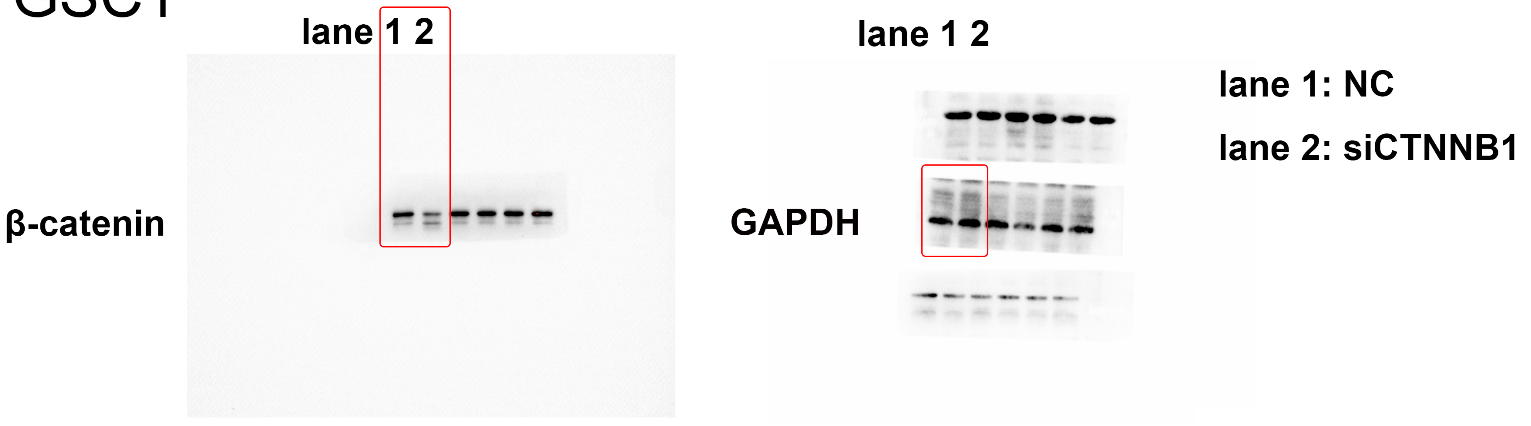


GSC11

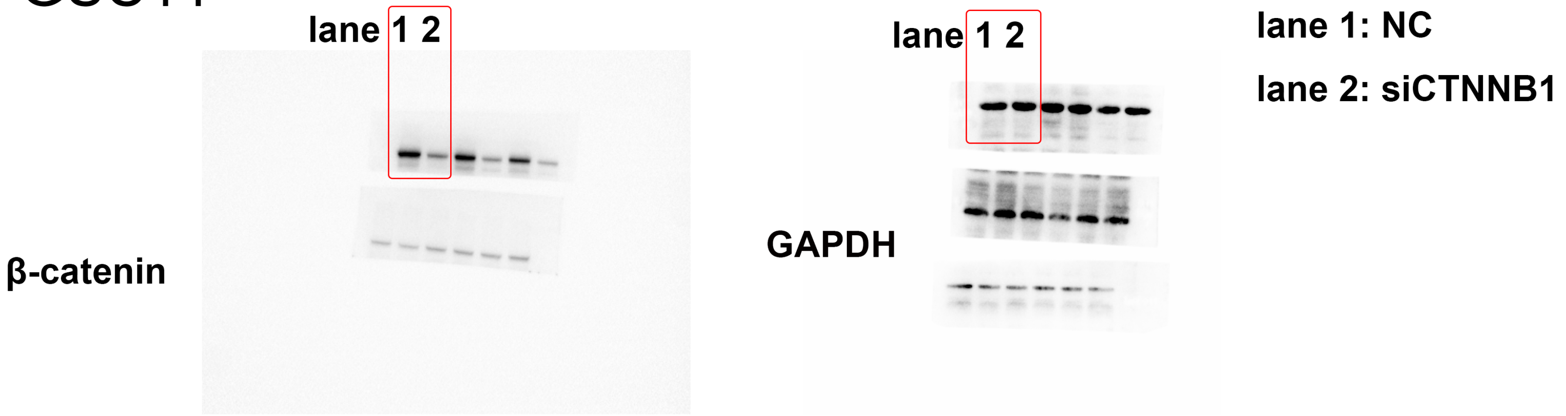

Supplement: Supplementary file 8 — Original Data of WB [file 41419_2022_4957_MOESM8_ESM.pdf]
